# Recovery of Repressed Memories in Fibromyalgia Patients Treated With Hyperbaric Oxygen – Case Series Presentation and Suggested Bio-Psycho-Social Mechanism

**DOI:** 10.3389/fpsyg.2018.00848

**Published:** 2018-05-29

**Authors:** Shai Efrati, Amir Hadanny, Shir Daphna-Tekoah, Yair Bechor, Kobi Tiberg, Nimrod Pik, Gil Suzin, Rachel Lev-Wiesel

**Affiliations:** ^1^Sagol Center for Hyperbaric Medicine and Research, Assaf Harofeh Medical Center, Zerifin, Israel; ^2^Sackler School of Medicine, Tel Aviv University, Tel Aviv, Israel; ^3^Sagol School of Neuroscience, Tel Aviv University, Tel Aviv, Israel; ^4^Ashkelon Academic College, Ashkelon, Israel; ^5^Social Work Department, Kaplan Medical Center, Rehovot, Israel; ^6^Department of Psychology, Loewenstein Hospital Rehabilitation Center, Raanana, Israel; ^7^Psychiatric Services, Assaf Harofeh Medical Center, Zerifin, Israel; ^8^The Emili Sagol CAT Research Center, Graduate School of Creative Arts Therapies, University of Haifa, Haifa, Israel

**Keywords:** hyperbaric oxygen, repressed memories, fibromyalgia, biopsychophysical mechanism, childhood sexual abuse

## Abstract

Fibromyalgia Syndrome (FMS) is a condition considered to represent a prototype of central sensitization syndrome, characterized by chronic widespread pain and along with symptoms of fatigue, non-restorative sleep and cognitive difficulties. FMS can be induced by trauma, infection or emotional stress with cumulative evidence that dissociation is relatively frequent in FMS patients. Two randomized controlled trials have shown that hyperbaric oxygen therapy (HBOT) can induce neuroplasticity and be effective in patients suffering from FMS. In this paper we present, for the first time, case series of female fibromyalgia patients who, in the course of HBOT, suddenly recalled repressed traumatic memories of childhood sexual abuse (CSA). The surfacing of the repressed (dissociative) memories decades after the sexual abuse events was sudden and utterly surprising. No psychological intervention was involved. As the memories surfaced, the physical pain related to FMS subsided. In one patient who had brain single photon emission CT (SPECT) before and after HBOT, the prefrontal cortex appeared suppressed before and reactivated after. The 3 cases reported in this article are representative of a total of nine fibromyalgia patients who experienced a retrieval of repressed memory during HBOT. These cases provide insights on dissociative amnesia and suggested mechanism hypothesis that is further discussed in the article. Obviously, prospective studies cannot be planned since patients are not aware of their repressed memories. However, it is very important to keep in mind the possibility of surfacing memories when treating fibromyalgia patients with HBOT or other interventions capable of awakening dormant brain regions.

## Introduction

Fibromyalgia Syndrome (FMS) is a condition characterized by chronic widespread pain and diffuse tenderness, along with symptoms of fatigue, non-restorative sleep and cognitive difficulties. It affects 2–5% of the general population worldwide, with 9:1 female-to-male incidence ratio (Buskila, 2009; Branco et al., 2010; Clauw et al., 2011; Schmidt-Wilcke and Clauw, 2011; Jones et al., 2015). FMS is considered to represent a prototype of central sensitization, i.e., a condition characterized by an increase in the transmission and processing of pain within the central nervous system (Yunus, 2007a,b; Efrati et al., 2015; Ablin et al., 2016). Like other functional somatic syndromes, despite suffering from pain affecting soft tissues (muscles, ligaments), and tendons, patients seem well without obvious abnormalities on neither physical examination nor evidence of tissue inflammation on laboratory and radiologic studies (Clauw, 2014).

In recent years, there are cumulative data regarding the complex biology of chronic pain within the CNS (Ablin et al., 2016; Sancassiani et al., 2017; Yavne et al., 2018). Chronic pain syndromes in general, and FMS in particular, are not congenital; these conditions rather evolve over lifetime, often in response to various external factors such as physical trauma, infection or emotional stress, which emphasizes the capability of the CNS to morph and re-wire during life, even at a fully developed stage (Ablin et al., 2016; Yavne et al., 2018). Hence, the general concept of neuro-plasticity is an essential pattern of chronic pain evolution.

In this study, our main focus is on FMS induced by childhood sexual abuse (CSA). CSA has negative long term physical and psychological effects and women with a history of CSA may develop depression, post-traumatic stress disorder (PTSD), dissociative disorders, and chronic pain syndromes such as FMS (Spiegel et al., 2015). Dissociation in CSA is particularly relevant to amnesia of all or part of the traumatic events (Bohn et al., 2013). Studies have shown that dissociation is relatively frequent in FMS patients (Naring et al., 2007; Bohn et al., 2013; Kilic et al., 2014; Berkol et al., 2017). Unfortunately, despite the fact that chronic pain syndromes are common in adult survivors of CSA, patients rarely reveal their abuse history to their care providers due to amnesia, dissociation, or merely shame, embarrassment or trust issues.

Hyperbaric oxygen therapy (HBOT), the application of hyperbaric pressure in conjunction with increased oxygen content, has been shown in several clinical studies to have the capacity to induce neuroplasticity in injured brains even years after an acute insult (Boussi-Gross et al., 2013, 2015; Efrati et al., 2013; Efrati and Ben-Jacob, 2014; Hadanny et al., 2015; Tal et al., 2015; Hadanny and Efrati, 2016). As demonstrated in different animal models, HBOT induces neuroplasticity by stimulating cell proliferation (Mu et al., 2013), promotes neurogenesis of endogenous neural stem cells (Yang et al., 2008), regenerates axonal white matter (Chang et al., 2009), improves maturation and myelination of injured peripheral and cranial neural fibers (Haapaniemi et al., 1998; Vilela et al., 2008), induces brain angiogenesis (Tal et al., 2015), and stimulates axonal growth (Mukoyama et al., 1975; Bradshaw et al., 1996).

Despite the fact that HBOT for neurological disorders is somewhat still controversial there is recent evidence of its effectiveness in treating FMS (Ablin et al., 2016). To date, two prospective randomized controlled trials have demonstrated the efficacy of HBOT in fibromyalgia (Yildiz et al., 2004; Efrati et al., 2015). The improvement was demonstrated in all aspects of FMS including pain threshold, fatigue, distress and quality of life (Yildiz et al., 2004; Hadanny et al., 2015).

In this paper we present three cases of women suffering from fibromyalgia, who recalled suppressed traumatic memories of CSA during HBOT. These cases provide additional important insights on memories retrieval and dissociative amnesia and the opportunity to suggest a possible mechanism hypothesis that is further discussed in the article. The participants in the current paper were not subject to any psychological intervention during the time of HBOT. As the memories surfaced, around the 20th HBOT session (with more memories surfacing as the treatment continued) the physical pain related to the FMS subsided. Following participants’ disclosure to a nurse, they were assigned to a physician for further assistance and investigation.

It should be noted that these events of self-disclosure were unfamiliar and unexpected for both patients and medical staff.

## Materials and Methods

A retrospective case presentation of FMS patients who reported retrieval of repressed memories during HBOT at the Sagol Center for Hyperbaric Medicine and Research at Assaf Harofeh Medical Center, Israel. The three cases reported in this article represent nine FMS patients who experienced the same during HBOT, out of a larger cohort of ∼200 FMS patients treated by HBOT in our center between 2013 and 2016. All reported patients, included in this article, signed an informed consent for their case publication. After patients signed consent, they reviewed and proofed their presented case. There were six additional patients with repressed memories recovery who did not agree for case publication. Thus, their cases are not presented. Vulnerable populations (for example minors, persons with disabilities) were not involved in this study. The publication process of those cases was approved by the head of Assaf Harofeh Medical Center Institutional Review Board.

The following HBOT protocol was practiced: 60 daily sessions, 5 days a week, 90 min each session, breathing 100% oxygen at 2.0 absolute atmospheres (ATA) with 5 min air breaks every 30 min. Sessions were performed in “multiplace” chambers, which may treat up to 12 patients simultaneously.

The diagnosis of FMS was based on the following criteria: (1) Symptoms of widespread pain, both above and below the waist and bilaterally. (2) Physical findings of at least 11 out of a set of 18 known tender points.

## Results

### Case Study 1

N, a 21 year-old female, was diagnosed at the age of seventeen with severe FMS, because of which she had to quit studying. N was a popular, good student, a third-born of three children to her highly educated parents. At the age of fourteen she insisted on moving to another school, rationalizing it as her simply wishing to study at a “better school.” Her parents agreed. She then invested all her energy in studying and in dancing lessons, in which she excelled until the onset of her fibromyalgia. N was referred to the Sagol hyperbaric institute by the family physician to receive HBOT for her fibromyalgia. Prior to her referral to HBOT she was treated with several different medications and with medical Cannabis for her severe pain. Between the 17 and the 34 HBOT sessions, N complained on low energy and tiredness. In additional, N told the medical staff that she feels sadder and more anxious than prior to HBOT. During that time, she started to experience flashbacks of herself being sexually molested. The flashbacks occurred both during HBOT sessions (inside the chamber) and in the hours following a HBOT session (at her normal environment). The flashbacks started with unbearable sexual arousal, accompanied with shortness of breath, burst into tears and other panic attack related symptoms such as feeling of loss of control and overwhelming fear. Fragments of memories appeared, in which she was being raped by several peers which added up to a full recovery of her repressed memory of being repeatedly molested and raped by classmates at the age of fourteen.

Looking back, N said: “I can’t understand how I, or anyone else for that matter, could forget such experiences… I continued to function as an excellent student; I focused on my dancing… I avoided peer gatherings, rationalizing this avoidance to myself and to my parents as “lack of interest” and “will to excel.”

N reported that, after the repressed memories emerged, she felt much stronger, slept better, had more energy and had almost no pain. According to N, she was fully recovered from her FMS.

### Case Study 2

A, a 56 year-old businesswoman, married with four children, the eldest daughter in her family of origin. As a child she had urolithiasis, for which she underwent bladder surgery three times. The relationships between her parents were complicated. Her mother used to have love affairs and brought her sexual partners home in the father’s absence. The mother also used to share her sexual experiences with A, who was a “parental child.” At the age of seven, A has been visiting an 18 year-old neighbor in his apartment three times a week and was receiving little presents from him. After the first visit, A cut her long, beautiful, blond hair without informing her parents, telling them later that she “preferred to be ugly.” About a year later, during the 6 days War, the family moved for a week to her grandmother’s. A describes this week as “the best, quietest, calmest week of my life, despite the fear of being at war.” Upon returning home, she refused her neighbor’s invitations and forgot his name. Shortly after graduation she got married. In 2011, she had a car accident and referred to treatment at the Sagol HBOT institute. Around that time she was also diagnosed with FMS. Between the 37th and 44th HBOT sessions, A experienced flashbacks of sexual molestation. The flashbacks were accompanied with anxiety and feelings of losing control in addition to sympathetic signs such as sweating. The flashbacks began with unbearable sexual arousal and concurrent panic attacks. Flashbacks initially occurred during the hyperbaric sessions, and later on happened during her daily routines between sessions. Visually vivid memories surfaced including her being ordered to have oral intercourse. These flashbacks gradually joined into detailed, coherent memories of sexual molestation she had experienced at the age of seven by her 18 year-old neighbor, who threatened to kill her if she ever revealed their “secret.” In an attempt to validate these memories, A returned to her childhood neighborhood, found the perpetrator, and confronted him. He confirmed that this had indeed occurred as remembered.

### Case Study 3

B, a 36 year-old female, was the youngest of three siblings in her family of origin. When admitted to HBOT for fibromyalgia, she reported having been sexually abused by her uncle (her mother’s brother) between the ages of six and sixteen. She said that her parents didn’t know about the abuse. “Actually,” she said “I think that, back then, I myself did not remember it most of the day, kind of detached.” Her mother died of cancer a year after the abuse ceased. B described her mother as “an innocent, good mother who knew nothing of what was going on.” In adulthood, B had psychotherapy for her CSA. During her Social Work studies, B began to suffer from fibromyalgia. The disease prevented her from committing to a full-time work as a social worker. At the age of thirty-six, she started HBOT for her FMS. During the first twenty sessions, she reported sexual arousal and unbearable pain in her abdomen, “felt as if someone was stabbing me with a knife.” These symptoms were followed by surfacing of new memories of the abuse revealing another abuser – her mother. “My mother took me to the shower after he [the uncle] abused me. She undressed me, ordered me to open my legs and then began to abuse my genitals.” These new, vivid, coherent, and detailed memories exposed her to different narratives on her mother and her childhood. Those memories of abuse by B’s mother did not surface prior to the HBOT, even during psychotherapy. After those new memories surfaced she returned to psychotherapy.

**FIGURE 1:**
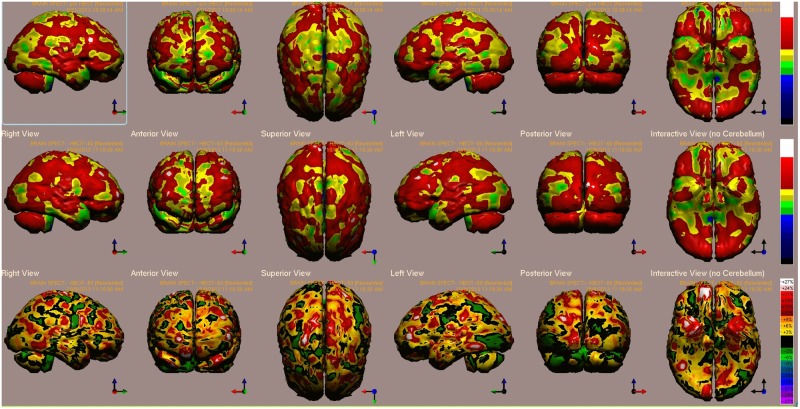
Case 1 single photon emission CT (SPECT) calculated change after hyperbaric oxygen therapy (HBOT) compared to baseline. In the **Top** two rows colors, represent maximal functional brain activity relative to the entire brain activity; in the **Bottom** row, colors represent regional changes in functional brain activity. White and red areas show the biggest relative changes in cerebral blood flow/perfusion. Significant changes can be seen at the right prefrontal and temporal areas (white). Abbreviations: Analysis of brain SPECT performed pre- and post HBOT showed the highest relative increase in activity (>15%) in the visual cortex (BA 19), prefrontal cortex (BA 9), orbito-frontal gyrus (BA 11), and paratemporal cortex (BA 37).

B’s baseline and post HBOT brain single photon emission CT (SPECT) scans are presented in **Figure 1**. The highest relative increases in activity (>15%) are seen in the visual cortex (BA 19), prefrontal cortex (BA 9), orbito-frontal gyrus (BA 11) and paratemporal cortex (BA 37) (**Figure 1**).

## Discussion

This article is the first report of retrieval of repressed memories of CSA in FMS patients during HBOT. In the three cases presented, HBOT was initiated for FMS, decades after the sexual abuse events occurred. The retrieval of the repressed memory through physiological intervention that induces neuroplasticity was unexpected. This phenomenon may augment our knowledge on the pathophysiological process related to dissociation and dissociative amnesia and suggest new physiological interventions to enhance the current psychological interventions.

### Dissociation, Dissociative Amnesia and Biopsychophyisical Mechanism

Trauma is a biopsychophysical experience, even when the traumatic event doesn’t cause direct mechanical harm to the body. According to this, the organism is defined by a collection of multiple biological and psychological interactions as well as social factors throughout its life (Bowman, 2011; Nijenhuis and van der Hart, 2011). The term “Dissociation” refers to the failure of integrating aspects of memory, identity, perception, and consciousness. Dissociative amnesia, is a failure of the ability to remember autobiographical information in the absence of brain damage (Staniloiu and Markowitsch, 2014). Thus “Dissociation” means that a memory is neither lost nor forgotten but is unavailable for retrieval for a period of time, which could last even decades (Chu et al., 1999). Dissociative amnesia or the suppression of traumatic memories, is well-documented in the literature and is coded as a dissociative disorder in Diagnostic and Statistical Manual of Mental Disorders (DSM-5^®^), Fifth Edition (American Psychiatric Association, 2013 and American Psychiatric Association. DSM-5 Task Force).

Individuals with dissociative disorders usually report a history of exposure to traumatic stress, development trauma, or other events that trigger stress known as stressors (Andrews et al., 1999; Bohn et al., 2013; Fergusson et al., 2013; Lev-Wiesel and Zohar, 2014). In psychological terms, dissociation may be considered as a protective mechanism to shield the traumatized individual from the unbearable pain of the memory and its consequences. CSA is an exceptionally severe form of emotional and physical trauma that may lead to disorders of memory and dissociation (Andrews et al., 1999; McNally, 2003; van der Kolk et al., 2005; Fergusson et al., 2013; Lev-Wiesel and Zohar, 2014). Several clinical studies demonstrate that CSA is associated with FMS severity and may shape the biological development of interoception in ways that predispose to pain and polysymptomatic distress (Romans et al., 2002; Ciccone et al., 2005; Jiao et al., 2015; Ortiz et al., 2016).

The classic fight-or-flight response to a perceived threat is a reflexive neural phenomenon which has obvious survival advantages in evolutionary terms (Sherin and Nemeroff, 2011). However, under certain circumstances, such as the CSA cases described above, exposure to a perceived threat can cause dysregulated response (Scaer, 2001; Sherin and Nemeroff, 2011). Chronic dysregulation can lead to functional impairment in certain individuals who become “psychologically traumatized” with long standing neurobiological abnormalities, which overlap features found in patients with post-concussion syndrome due to traumatic brain injury (TBI) (Scaer, 2001; Sherin and Nemeroff, 2011). Certain brain areas become disconnected from the normal trophic stimulations of cerebral perception and subjected to the extremes of vasomotor instability of trauma that may lead to pathologic vasoconstriction and ischemia (Scaer, 2001). This model explains the somatoform dissociative symptomatology that may arise in TBI or CSA, followed by FMS. Thus, FMS can be induced by either TBI or CSA.

### Suggested Physiological Mechanism for HBOT Effect in Dissociation

From the neurobiological/physiological perspective, the authors suggest a possible mechanism for the recovery of repressed memories induced by HBOT.

**FIGURE 2:**
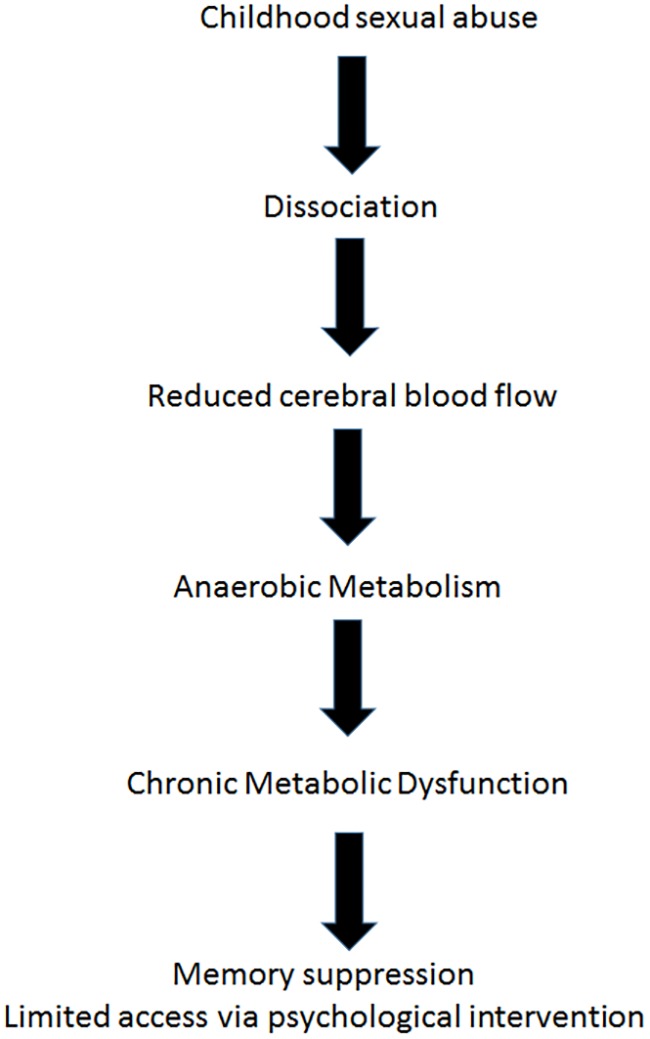
Dissociation and repressed memory – suggested model for the neuropathology cascade.

Astrup et al. (1977), demonstrated in a paper argued to be one of the most important in applied neurology, that after the onset of focal ischemia (such as blood vessel occlusion), measurements of electrical activity reveal brain regions that are dysfunctional but still viable (Astrup et al., 1977). The neurons in this area retained enough energy to maintain ion pumps and sustain the -70-mV resting membrane potentials required for their existence. However, those neurons did not have enough energy to generate the action potentials needed for their purpose functioning. In the last decade, functional imaging of the brain by positron emission tomography (PET) and SPECT scanning (Heiss, 2000; Baron, 2001; Siddique et al., 2002; Ebinger et al., 2009; Meerwaldt et al., 2010), afforded us images of such areas, and the accumulating data indicate that apparently dead areas may persist for years after an acute event (Boussi-Gross et al., 2013; Efrati et al., 2013). The stunned/hibernating areas, unable to fire action potentials, are characterized by metabolic dysfunction, namely anaerobic metabolism and ATP depletion (**Figure 2**). Loss of ATP and the resultant intracellular acidosis would bring about energetic breakdown of ion pumps and, consequently, additional damage to the mitochondria and endoplasmic reticulum (Hossmann, 2006; Culmsee and Krieglstein, 2007). Furthermore, anaerobic metabolism would bring about elevation of free radical levels (Hossmann, 2006; Culmsee and Krieglstein, 2007), persistent inhibition of protein synthesis (DeGracia, 2004), selective neuronal damage demonstrable by decreased density of benzodiazepine and/or 5-HT2 receptors (Vera et al., 1996; Yamauchi et al., 2005), augmented intracellular calcium (Hossmann, 2006), blood-brain barrier damage and augmented inflammation evidenced by higher levels of cytokines and cyclooxygenase-2 (COX-2) (Yin et al., 2002).

The brain regions with continuous hypoperfusion, with their very low, anaerobic metabolic rate, settle into a quasi-steady energy well from which they cannot climb up on their own. The hibernation state typical of some mammalian species seems to be the most proper model created by nature, simulating the post injury and post ischemia states of brain (Drew et al., 2007). The energy consumption in hibernation is less than 0.1 WT/kg, i.e., less than 1% of the metabolism in a resting state. The corresponding outcomes at the cellular level include a dramatic (∼2500-fold) decrease in protein synthesis (Frerichs et al., 1998), complete interruption of neuronal spike activity (Carey et al., 2003), and a switch from carbohydrate-based to fat-based metabolism (Carey et al., 2003). In turn, metabolic changes induced by minimized levels of energy availability have an impact on brain structure and functioning at the cellular level, such as reorganization of cell membranes, formation of protein-free domains that displace membrane proteins, and altered cell membrane permeability due to alterations in cytoplasmic matrix (Azzam et al., 2000; Adibhatla and Hatcher, 2008).

The neurobiological mechanism of dissociation, as mentioned above involves disconnection of certain brain areas, may be explained by hibernation, a hypometabolic state at the cellular level (minimal energy generation by the mitochondria in order to maintain membrane potential for cell survival). Hibernated areas may correlate with apathy and low responsiveness (Schore, 2009). This model supports the assumption that dissociation could be interpreted from the biological perspective as a hypometabolic state and from the biopsychological perspective as a deficiency of psychological energy. In the cases presented, the metabolic dysfunction of the dissociated brain regions serves as a physiological boundary for psychological intervention (**Figure 2**).

**FIGURE 3:**
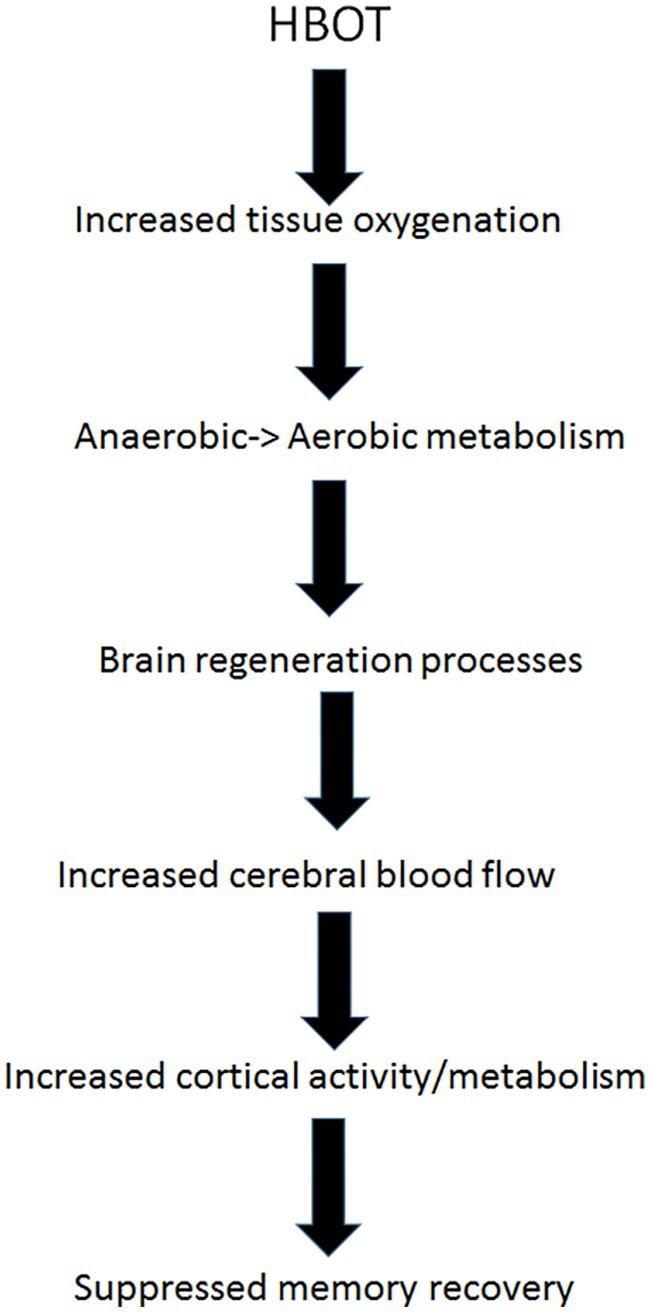
Recovery of repressed memory by HBOT – suggested mechanism.

Energy wise, climbing back up from the “metabolic well” mandates energy input. As discussed above, HBOT has been shown to induce neuroplasticity and reactivation of cells in chronic metabolic dysfunction in different type of brain injuries, and in FMS specifically (Mukoyama et al., 1975; Bradshaw et al., 1996; Haapaniemi et al., 1998; Yildiz et al., 2004; Vilela et al., 2008; Yang et al., 2008; Chang et al., 2009; Boussi-Gross et al., 2013, 2015; Efrati et al., 2013; Mu et al., 2013; Efrati and Ben-Jacob, 2014; Hadanny and Efrati, 2015, 2016; Hadanny et al., 2015; Tal et al., 2015). HBOT supplies extra oxygen to the anaerobic brain regions, providing them with the energy needed to exit metabolic well. Once these regions of metabolic dysfunction are re-activated by HBOT, cerebral blood flow is increased to these regions and they return to normal metabolism. Hibernating brain regions which may have been responsible to memory repression, can be reactivated by HBOT, regain their normal function and resurface suppressed memories (**Figure 3**).

### Potential Role of Prefrontal Cortex Role in Suppressed Memories

One of the patients (Case 1) presented in this article had brain SPECT before and after HBOT (**Figure 1**). In this case, the prefrontal cortex that was suppressed at baseline was reactivated by the use of HBOT.

Repression of memories, or dissociative amnesia, has been correlated with decreased activity of the dorsomedial prefrontal cortex, while recovery is correlated with increased prefrontal cortex activity as shown by functional magnetic resonance imaging (fMRI) (Anderson et al., 2004; Kuhl et al., 2008; Che et al., 2015). PET studies in patients suffering from PTSD demonstrated increased cerebral blood blow in the prefontral cortex when re-experiencing emotionally charged episodic memories (Masaki et al., 2006; Im et al., 2016).

### Study Limitations

This article has several obvious limitations. Most limitations are related to the small number of patients and the lack of brain SPECT imaging in the other two patients. Another limitation relates to the fact that there is no known way to fully and objectively validate the retrieved memories. Still, two of the women confronted their past perpetrators, who begged for their forgiveness. The three cases presented in this article are representative of 9 women who had retrieved suppressed memories during HBOT at our center. In order to gain better understanding of dissociation/dissociative amnesia and its suggested biopsychophyisical mechanism and long term biological consequences, direct evaluation of brain metabolism/activity in animal models should be investigated.

## Conclusion

This is the first clinical report of recovery of suppressed memories with HBOT alone as physiological intervention. Even though further studies are needed, it would not be easy to carry out a study in patients that are unaware of their difficult childhood history. Obviously, prospective studies cannot be planned since patients are not aware of their repressed memories. However, it is very important to keep in mind the possibility of surfacing memories when treating FMS patients with HBOT or other interventions capable of awakening dormant brain regions. We recommend that HBOT practitioners treating FMS patients should add the potential risk for unexpected memory of traumatic events to occur to their informed consent. All medical professionals who are dealing with FMS patients should be aware and prepared to handle such outcome. It would be highly beneficial if anyone who encounters such a case documents and reports it. Through the cumulative data we may get important insight into the neurobiology of suppressed memories.

## Author Contributions

SE: study initiator, interpretation of study results, and wrote the first and final drafts of the manuscript. AH: interpretation of study results, brain Image analysis, and co-wrote with SE first and final drafts of the manuscript. SD-T: interpretation of study results, data collection, patients follow up, and revision of the manuscript. YB: data collection and patients follow up. KT: interpretation of study results and revision of the manuscript. NP: patients follow up and interpretation of study results. GS: interpretation of study results and revision of the manuscript. RL-W: patients follow-up, interpretation of study results, and revision of the manuscript. All authors read and approved the final manuscript.

## Conflict of Interest Statement

The authors declare that the research was conducted in the absence of any commercial or financial relationships that could be construed as a potential conflict of interest.
